# Cosmetic after-feel modulates brain activity in sensory and reward networks: an fMRI study

**DOI:** 10.3389/fnins.2026.1759372

**Published:** 2026-03-10

**Authors:** Audrey Maniere, Arnaud Pêtre, Ron Kupers, Céline Manetta, Joan Attia, Eloïse Gerardin

**Affiliations:** 1 Lucas Meyer Cosmetics, Massy, France; 2 Brain Impact Neuroscience, Chemin du Bois Magonette, Lasnes, Belgium; 3 HE-Arc Gestion, Institut de la Communication et du marketing expérientiel (ICME), Neuchâtel, Switzerland; 4 Institute of NeuroScience (IoNS), UCLouvain, Brussels, Belgium; 5 International Flavors and Fragrances, Neuilly Sur Seine, France

**Keywords:** affective touch, after-feel, fMRI, reward network, somatosensory processing

## Abstract

The affective dimensions of cosmetic textures were investigated using functional magnetic resonance imaging (fMRI) to examine how after-feel, defined as residual tactile sensations persisting on the skin after product application, modulates sensory and emotional processing. Twenty healthy women took part in three conditions: no cream (control), cream A, or cream B, differing only in emulsifier composition. A fixed amount of cream was applied to predefined areas of the left hand. After absorption, participants stroked these areas at a controlled speed. fMRI data were acquired during this self-touch task, preprocessed using a standardized pipeline, and analyzed using a general linear model. Results showed that the no-cream and cream B conditions primarily engaged primary somatosensory regions, consistent with basic tactile encoding. In contrast, cream A additionally recruited brain areas involved in affective and reward processing, including the orbitofrontal cortex, amygdala, and putamen, with key reward-related responses, notably within striatal and insular regions, showing a right-hemispheric dominance contralateral to the hand receiving the tactile input. This broader activation pattern suggests that specific cosmetic ingredients can enhance the emotional salience of after-feel, potentially through C-tactile afferent pathways mediating affective tactile signals. These findings reflect a hierarchical integration of tactile input, from sensory encoding to higher-order affective appraisal. They highlight the potential of cosmetic formulations to influence central touch representation beyond surface-level sensation. This proof-of-concept study offers novel insights into how the sensory and emotional qualities of cosmetic products take shape in the brain, providing a neuroscientific foundation for the development of emotionally engaging textures.

## 1 Introduction

The sense of touch plays a central role in the experience of cosmetic product use, as applying a cream inherently involves self-directed tactile stimulation across multiple phases of interaction, including initial contact with the product, the application movement itself, and the residual sensations that persist after application (Krishna et al., 2024). This self-touch engages not only primary somatosensory cortices but also brain regions involved in emotional processing and salience attribution, such as the insula, anterior cingulate cortex, and orbitofrontal cortex (McGlone et al., 2014; Morrison, 2012; Ebisch et al., 2011), which play a key role in the affective evaluation of tactile input and in assigning emotional relevance to bodily sensations during product use. Although texture perception involves multiple sensory modalities, touch appears to be particularly critical in shaping how cosmetic products are experienced (Duncan et al., 2020; Courrèges et al., 2021; Wang et al., 2024).

Beyond the act of touch itself, cosmetic products generate a second and distinct sensory phase, referred to the so-called after feel, defined as the persistent sensation that remains on the skin once the product has penetrated and dried (de Noronha et al., 2019). While self-touch has been extensively examined in affective neuroscience, cosmetic after-feel remains largely unexplored, despite its relevance to consumer experience (Shirata and Campos, 2016; Baki et al., 2018). Unlike self-touch, after-feel is not driven by active movement but by formulation properties and the residual film, and manifests as distinct tactile qualities such as smoothness, stickiness, or greasiness (Akanny and Kohlmann, 2024). For example, oils create a lasting slippery effect (Savary et al., 2019), whereas powders modulate smoothness depending on their shape, size, and composition (Moussour et al., 2017). This phase of the cosmetic usage experience has been extensively characterized using sensory panels and instrumental methods but has rarely been examined from an emotional perspective (Wang et al., 2024).

Given that after-feel sensations directly contribute to emotional appraisal, emotional responses to tactile experiences during cosmetic use can be objectively investigated using physiological and neuroimaging approaches. For example, increased activation in the right dorsolateral prefrontal cortex during cosmetic use, as measured by functional near-infrared spectroscopy, has been associated with a greater willingness to pay (Duncan et al., 2019). Complementary evidence from electroencephalography (EEG) studies has captured real-time emotional responses during the application of creams or lip balms (Gabriel et al., 2021; Lombardi, 2017). These findings highlight the influence of specific ingredients, such as thickening agents or emollients, on the emotional valence associated with cosmetic use. Although EEG signals have been shown to correlate with self-reported valence, they appear less sensitive to differences in arousal across products (Wang et al., 2024).

While existing fMRI studies have primarily focused on pleasant touch in non-cosmetic contexts, they consistently implicate brain regions involved in both reward processing and social cognition, supporting dedicated neural pathways for affective tactile processing (Gordon et al., 2013; Perini et al., 2015). Pleasant touch engages the orbitofrontal cortex, putamen, and pregenual anterior cingulate cortex, which code for reward value and hedonic experience (Sailer et al., 2016; Lindgren et al., 2012; Rolls et al., 2003). In parallel, regions including the temporoparietal junction, supramarginal gyrus, precuneus, and medial prefrontal cortex support social-cognitive processes such as empathy, perspective-taking, and self-referential thinking (Kogler et al., 2020; Alcalá-López et al., 2024).

Querleux et al. (1999) reported that the application of a cosmetic product enhances sensory cortical responses compared to bare skin stimulation, although emotional dimensions were not investigated. More recently, Hirao et al. (2020, 2021) showed that contextual cues, such as labeling a cream as *luxury*, increase activation in reward and social cognition networks, showing that both texture and perceived value contribute to the affective processing of cosmetic touch. The affective dimension of touch is supported by distinct mechanosensory systems. Fast-conducting A-β fibers are responsible for discriminative touch, conveying fine spatial and textural details (Johnson, 2001; Abraira and Ginty, 2013), while emotional aspects are mediated by unmyelinated C-tactile (CT) afferents, which respond preferentially to slow, gentle stimulation and are associated with pleasantness (McGlone et al., 2014; Marshall and McGlone, 2020). These parallel pathways contribute jointly to the perception of both the physical properties and emotional valence of touch. Although well characterized in social and experimental settings, this dual processing system has only recently been investigated in the context of cosmetic textures (Spence and Zhang, 2024). Within this framework, CT fibers, especially from hairy skin, project to the posterior insula, cingulate and prefrontal cortex, regions involved in affective valence processing (Löken et al., 2009; Rolls et al., 2003; McCabe et al., 2008; Gordon et al., 2013). This dual system has recently been considered in studies of cosmetic textures, suggesting that product-induced touch may engage both sensory and emotional brain networks.

Building on this idea, the present proof-of-concept study tests the hypothesis that the final state of the system formed by a cream and the skin surface after application modulates the neural processing of affective touch. Here, the final state of the cream refers to the components remaining on the skin after evaporation, absorption, or molecular conformational changes, while the final state of the skin surface encompasses changes in the stratum corneum induced by cream absorption, including its mechanical properties and surface texture. Together, these changes constitute the after-feel of the cream, which we propose directly influences the perceived pleasantness of self-touch by engaging brain regions involved in affective tactile coding.

## 2 Materials and methods

### 2.1 Participants

20 healthy female subjects (18–45 years of age; mean age: 31.5 ± 6.5 years; range: 19–45 years) participated in the study. All participants were right-handed. All participants completed a medical questionnaire to confirm eligibility. The study was approved by the ethics committee of the CHU UCL Saint-Luc (Brussels) (ethics approval number N°B403201112591), and all participants provided written informed consent prior to participation.

### 2.2 Experimental conditions

Three experimental conditions were evaluated: control (no cream), and two cosmetic creams (A and B.) with identical compositions, but differing in the emulsifier used. Both emulsions were fragrance-free and showed similar physical properties, including appearance (white and fluid), pH, and viscosity. The % of each emulsifier was chosen according to the formulation guidelines needed to emulsify 20% of the oil. Cream A was emulsified using modified starch particles, named S*odium starch octenyl succinate* derived from Quinoa, showing a narrow size distribution with a mean diameter of 2μm. Cream B (reference formulation) was emulsified using a traditional emulsifier composed of glyceryl stearate and *PEG-100 stearate*, widely used in commercial cosmetic products for decades (Supplementary Table 1).

### 2.3 Experimental design

**FIGURE 1:**
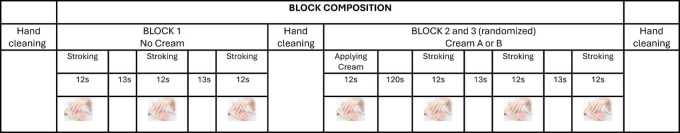
Experimental design and block description. The fMRI session included three blocks: cream-free massage, and massage with cream A and cream B (order randomized). Before each block, the hands were cleaned. In cream conditions, 80 μL was applied to the left hand and massaged for 12 s. After a 2-min rest, participants stroked the area three times at controlled speed to assess the after-feeling. The session ended with an anatomical scan.

Before the study began, participants received explanations and training outside the scanner to ensure the correct application of the cream in terms of movement trajectory and speed (Supplementary Image 1). Inside the MRI scanner, participants viewed a visual image of their hand projected onto a screen via the mirror system, which allowed them to monitor their hand position and maintain the instructed rhythm and trajectory throughout the task. Each active phase was explicitly cued by auditory beeps delivered through the scanner headphones, marking both the onset and the end of the movement period. Participants completed a single experimental session (∼1 h) consisting of structured phases: first explanations, training, an anatomical scan and fMRI acquisitions. The fMRI session included three sequential blocks: cream-free massage (e.g., no cream, Block 1), massage with cream A (Block 2), massage with cream B (Block 3); the order of the blocks was randomized across participants. Before each block, hands were cleaned with a remover wipe, rinsed with a wet cotton pad, and dried with a tissue, followed by a 2-min rest. In Blocks 2 and 3, 80 μL of cream was applied to the back of the left hand. Participants massaged each area for 12 s (6 rotations, ± 2 s/rotation) to make the cream penetrate the skin, followed by a 2-min rest. To assess the after-feel sensory effects, participants stroked their left hand with their right hand at a speed ( ± 2 s/rotation) three times with 13 s rest intervals. The session concluded with a 3D T1-weighted anatomical scan (Figure 1).

### 2.4 MRI data acquisition

The MRI data were acquired using a 3T GE SIGNA Premier scanner at UCL with a 2D echo-planar imaging (EPI) sequence. Characteristics of the fMRI data were repetition time (TR) 1.5 s echo time (TE) 30 ms, flip angle (FA): 90 degrees, FOV: 220 mm, with a matrix size of 110 × 110. The slice thickness was 2 mm with an interslice gap of 2 mm. Multiband acceleration was set to a factor of 3 with an in-plane parallel imaging acceleration factor of 2. The phase encoding direction was j- with an effective echo spacing of 0.00066 s and a total readout time of 0.07194 s. The pixel bandwidth was 4545.45 Hz. The specific absorption rate was 0.149036. High-resolution structural images were acquired using a 3D T1-weighted sequence on a 3T GE SIGNA Premier scanner at UCL. Parameters were TR = 2.188 s, TE = 2.96 ms, TI = 0.9 s, flip angle 8°, FOV 256 mm, matrix 256 × 256, slice thickness 1 mm, 1 mm spacing, parallel imaging factor 1.75, and pixel bandwidth 244.14 Hz (SAR 0.067). Images were acquired head-first supine with a 48-channel coil and used for registration of functional data to standard space.

### 2.5 Data pre-processing

Brain imaging data were preprocessed using fMRIPrep (nipreps.org), a standardized pipeline for task-based fMRI (Esteban et al., 2019). Preprocessing steps included slice timing correction based on a repetition time (TR) of 1,500 ms, and motion correction by rigidly aligning all functional volumes to the first volume of each session. Quality control confirmed that head motion did not exceed 3 mm of translation or 2° of rotation. A high-pass filter removes low-frequency drifts below 0.0063 Hz, and spatial smoothing with a 5 mm Gaussian kernel enhanced signal-to-noise ratios. Functional data were aligned to each participant’s native anatomical space using positional header information and gradient-based optimization. Anatomical data were normalized to the standard MNI T1 template using the “a12” transformation matrix, and functional data were resampled to a voxel resolution of 2 × 2 × 2 mm^3^ within the same space.

### 2.6 Data analysis

Brain imaging data were analyzed using a general linear model (GLM) to test voxel-wise differences in parameter estimates (beta weights) associated with the three experimental conditions, including no cream massage as a control condition, cream A and cream B. The analysis was conducted using a two-level GLM framework. At the first level, simple contrasts were computed to examine the neural response to each condition independently, including cream A versus baseline, cream B versus baseline, and no-cream versus baseline. At the second level, direct comparisons between conditions were made, including cream A versus no-cream, cream B versus no-cream, and cream A versus cream B. Group-level statistical maps were thresholded using a False Discovery Rate (FDR) correction at *q* < 0.05 to control multiple comparisons. Given the sample size (*N* = 20), sensitivity estimates for second-level one-sample *t*-tests (α = 0.05, power = 0.80) indicate that the analyses are primarily powered to detect medium-to-large effects (approximately Cohen’s d ≈ 0.63), such that small effects may not reach significance. All reported coordinates are expressed in Montreal Neurological Institute (MNI) space.

## 3 Results

### 3.1 Simple contrasts

**FIGURE 2:**
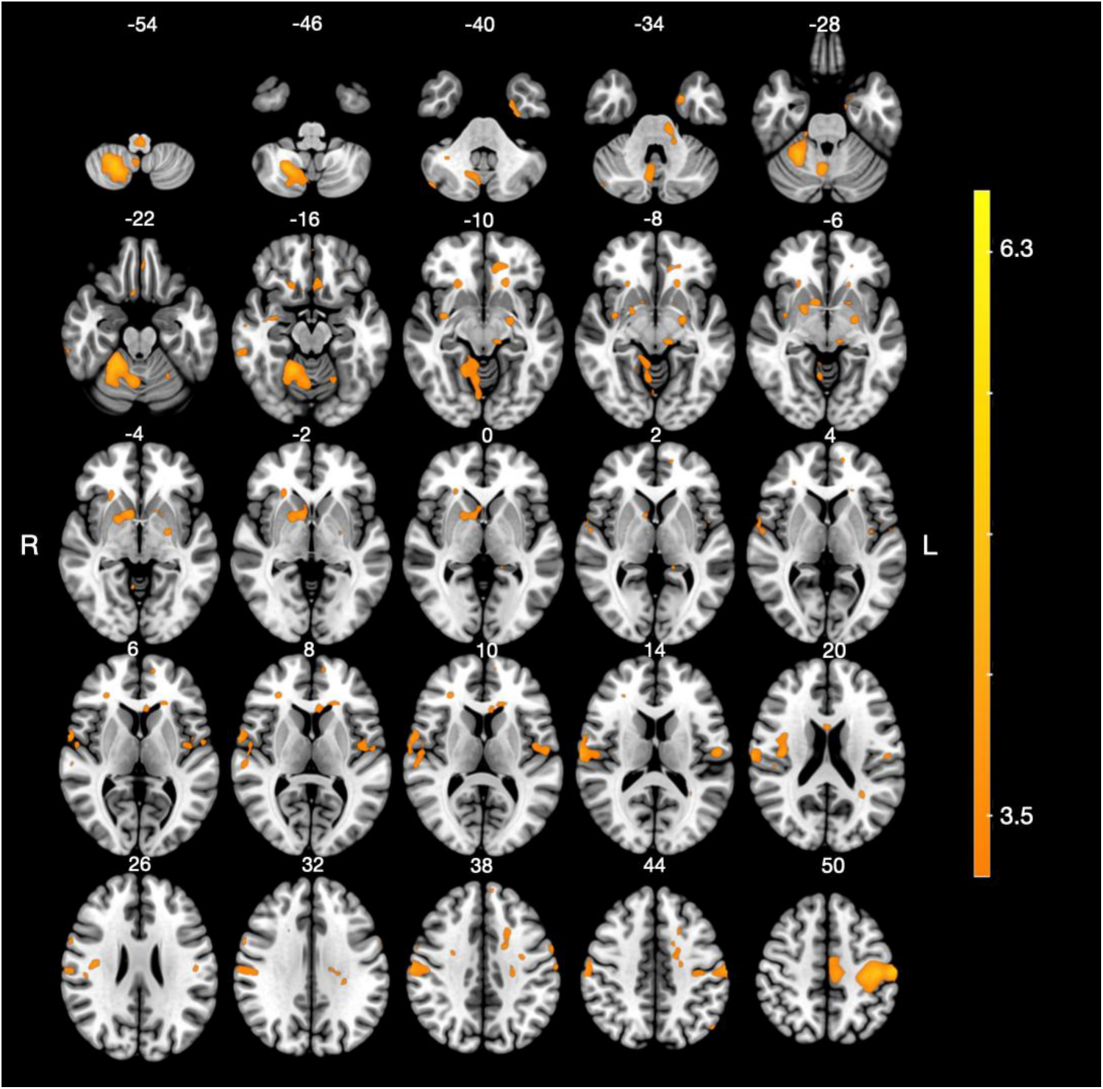
Brain activation map for the contrast *cream A > baseline*. Statistical parametric map showing brain activation associated with cream A application. The image displays axial slices at multiple *z*-coordinates ranging from *z* = –54 mm to *z* = 50 mm in MNI space. The color scale stands for *t*-values from the group-level analysis. Activations are observed primarily in somatosensory cortical regions, as well as in subcortical areas including the amygdala and nucleus accumbens. Images are displayed in radiological convention, with the left hemisphere shown on the right.

The GLM analysis revealed significant activations for the “no cream” condition in the left postcentral gyrus and the right cerebellum (see Supplementary Figure 1 and Supplementary Table 2). For the “cream A” condition, similar activations were observed in the left postcentral gyrus and the right cerebellum (Figure 2 and Table 1). Additionally, significant main effects were identified in the right postcentral gyrus, supramarginal gyrus, left precentral gyrus, and right nucleus accumbens. Other activated regions included the middle fronto-orbital gyrus, entorhinal area, gyrus rectus, and amygdala (Figure 2). For cream B condition, the largest activation was observed in the left postcentral gyrus, with additional significant clusters in the right cerebellum and the left precentral gyrus (Supplementary Table 3 and Supplementary Figure 2).

**TABLE 1:** Brain regions showing significant activation for the contrast cream A > baseline.

Structures	Size	Side	MNI coordinates			*t*-stat
			*x*	*Y*	*Z*	
Postcentral gyrus	15,146	L	−50	−19	60	6.3
	569	R	42	15	23	4.5
	299	L	−56	−13	12	4.1
	275	R	32	−29	62	3.8
Cerebellum	11,768	R	21	−61	−50	5.2
Supramarginal gyrus	1,975	R	66	−17	16	4.7
Precentral gyrus	1,581	L	−4	−16	56	4.1
Nucleus accumbens (ventral striatum)	4,903	R	10	9	−4	4.2
Middle fronto-orbital gyrus	211	R	20	25	−12	4.1
Entorhinal area	185	L	−21	0	−32	4.5
Gyrus rectus	178	L	−6	23	−14	3.8
Amygdala	161	L	−24	−7	−6	3.9

R, right hemisphere; L, left hemisphere; t, *t*-value. The table reports the number of voxels per cluster, MNI coordinates (x, y, z) of the peak voxel, and corresponding maximum *t*-values. All regions listed showed significant positive BOLD responses compared to baseline.

### 3.2 Complex contrasts

**FIGURE 3:**
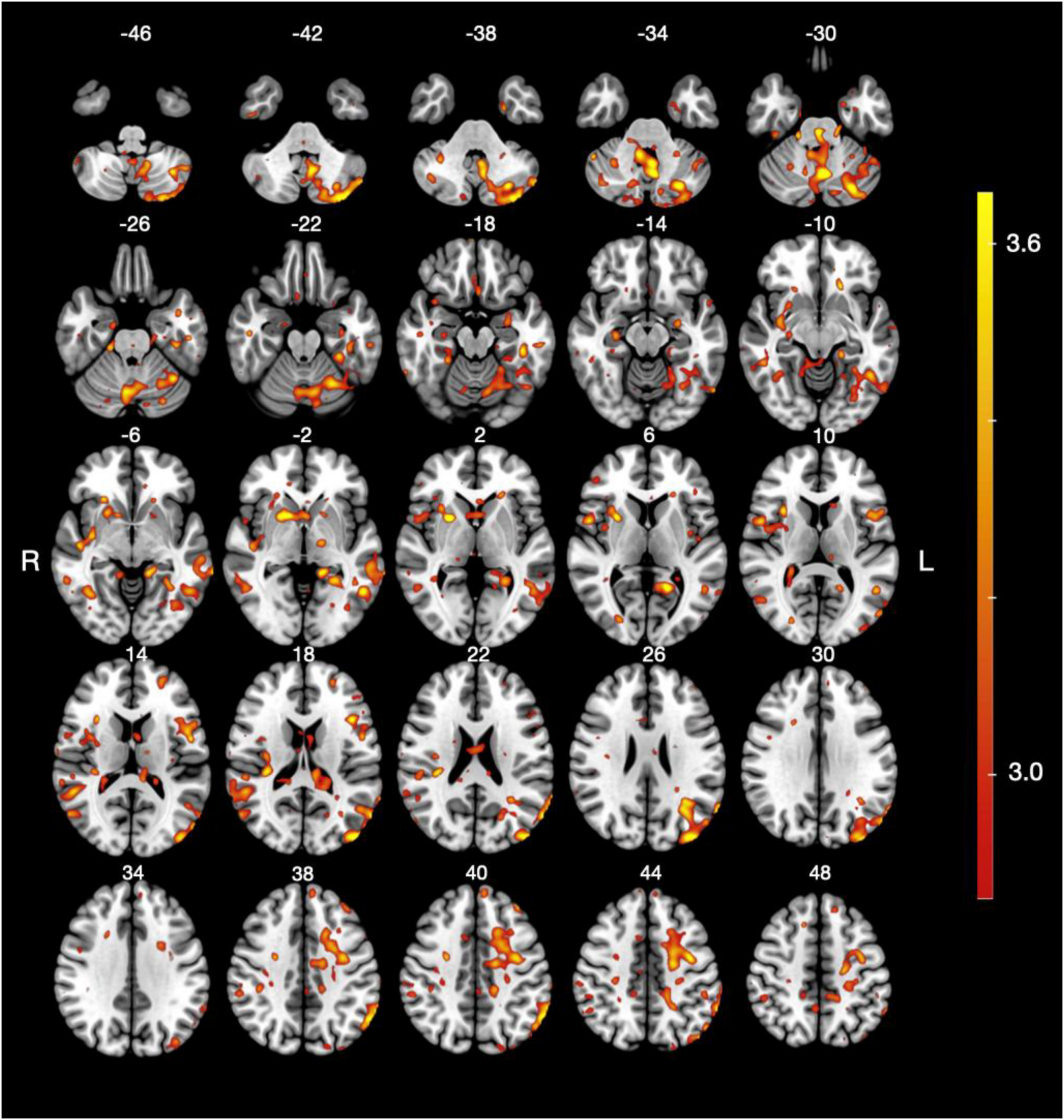
Brain activation map for the complex contrast *cream A > no cream*. Statistical parametric map showing significant BOLD signal changes for the cream A condition relative to the no cream condition. Axial slices are displayed at multiple MNI z-coordinates (*z* = –46 to 48). The color scale indicates *t*-values from the group-level analysis, thresholder at *p* < 0.05 (FDR-corrected).

The group-level GLM analysis comparing the cream A and no cream conditions (Figure 3 and Table 2) revealed significantly greater activations for the cream A condition in the left cerebellum and the left precentral gyrus. Additional extensive clusters were observed in the left middle occipital gyrus, right putamen, and the left inferior frontal gyrus (pars triangularis). Bilateral activations were also found in the angular gyrus, as well as in the temporal gyrus, including the left posterior middle and the right superior regions. Smaller clusters of activation were identified in the right insular cortex and the posterior insula.

**TABLE 2:** Brain regions showing significant activation for the complex contrast cream A > no cream.

Structures	Size	Side	MNI coordinates			*t*-stat
			*x*	*y*	*z*	
Cerebellum	23,501	L	−4	−63	−31	4.5
	2,015	L	−26	−65	−58	4.2
	288	R	40	−67	−38	3.3
	227	R	46	−49	−48	3.4
	196	R	11	−43	−8	3.3
	191	R	20	−59	−52	3.5
	114	L	−22	−49	−18	3.2
	112	R	25	−41	−18	3.4
	103	R	14	−71	−34	3.2
Precentral gyrus	4,925	L	−38	−7	44	3.7
	718	R	52	5	8	3.8
	409	R	36	1	16	3.3
	183	R	26	−5	40	3.6
	102	L	−12	−21	71	3.2
Middle occipital gyrus	4,468	L	−46	−87	18	4.3
	508	R	56	−67	16	3.5
	205	R	25	−83	8	3.5
Putamen	2,469	R	26	7	4	4.4
Inferior frontal gyrus pars triangularis	1,711	L	−43	17	17	3.8
Angular gyrus	1,327	L	−64	−49	42	4.1
	1,282	R	52	−47	16	3.7
	100	R	56	−39	38	3.2
Posterior middle temporal gyrus	1,308	L	−72	−41	−4	3.9
	502	R	52	−53	0	3.4
Superior temporal gyrus	1,197	R	46	−17	−4	3.8
Superior parietal gyrus	867	L	−18	−35	40	3.4
Postcentral gyrus	747	L	−26	−33	51	3.5
	205	R	14	−27	52	3.3
Fusiform gyrus	644	L	−42	−33	−18	3.7
	425	R	38	−59	−6	3.5
	274	L	−32	−37	−22	3.6
Supramarginal gyrus	559	R	34	−30	22	3.7
	263	R	52	−33	20	3.3
	106	R	40	−33	37	3.3
Precuneus	332	R	5	−47	52	3.1
Middle frontal gyrus	290	L	−26	51	16	3.4
	125	L	−38	39	40	3.8
Insula	275	R	25	25	−6	3.6
Inferior occipital gyrus	239	L	−60	−67	−14	3.6
Amygdala	223	L	−28	−7	−16	3.6
Superior frontal gyrus	193	L	−8	54	42	3.3
Posterior insula	187	R	34	−15	20	3.4
Posterior inferior temporal gyrus	182	R	50	−43	−10	3.4
	139	L	−58	−27	−20	3.4
Gyrus rectus	143	L	−2	19	−17	3.3
Entorhinal area	136	R	14	−9	−27	3.3
Inferior temporal gyrus	127	R	48	−16	−22	3.4
Superior occipital gyrus	119	L	−22	−69	22	3.2

The table reports cluster size (in voxels), hemisphere (L, left; R, right), MNI coordinates (x, y, z) of the peak voxel, and corresponding *t*-values (*t*-stat).

**FIGURE 4:**
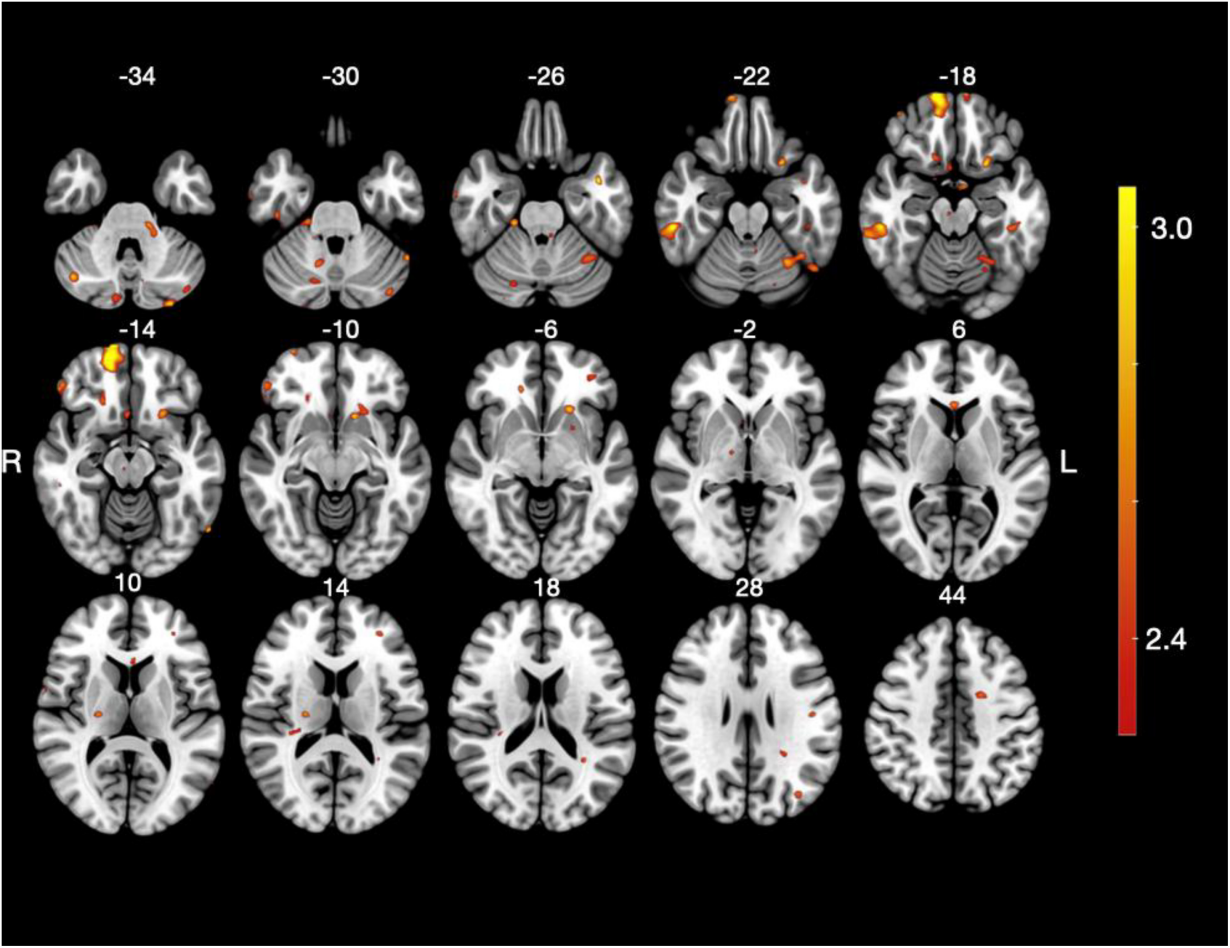
Brain activation map for the complex contrast *cream A > cream B*. Statistical parametric map showing brain regions with greater activation in the cream A condition compared to cream B. Axial slices are presented at multiple MNI z-coordinates (–35 mm to 43 mm). The color scale stands for *t*-values from the group-level analysis, thresholded at *p* < 0.05 (FDR-corrected).

When comparing the cream, A and cream B conditions (Figure 4 and Table 3), several clusters showed significantly greater activation for cream A. The largest activation was observed in the right middle fronto-marginal gyrus. Additional clusters were found in the right and left posterior inferior temporal gyri. Frontal regions with increased activation included the left lateral fronto-orbital gyrus, the right inferior frontal gyrus (pars orbitalis), the subgenual anterior cingulate gyrus, the left superior frontal gyrus, and the right middle fronto-orbital gyrus. Bilateral cerebellar activations were also identified.

**TABLE 3:** Brain regions showing significant activation for the complex contrast cream A > cream B.

Structures	Size	Side	MNI coordinates			*t*-stat
			*x*	*y*	*z*	
Middle fronto-marginal gyrus	1,980	R	14	67	−17	3.7
Posterior inferior temporal gyrus	1,100	R	54	−32	−20	3.4
	218	L	−46	−31	−20	2.8
Cerebellum	1,029	L	−37	−55	−24	2.9
	586	L	−40	−81	−30	2.7
	488	R	10	−89	−42	2.9
	276	R	20	−29	−26	3.0
	254	R	42	−69	−36	3.0
	221	L	−30	−89	−36	3.1
	182	R	14	−72	−32	2.7
	128	R	12	−59	−30	2.8
Lateral fronto-orbital gyrus	859	L	−24	15	−20	3.1
Superior frontal gyrus	592	L	−20	−1	42	3.1
Inferior frontal gyrus pars orbitalis	340	R	52	39	−12	3.1
Subgenual anterior cingulate gyrus	247	R	5	15	−20	2.8
Middle fronto-orbital gyrus	232	R	20	29	−12	2.9
Middle occipital gyrus	198	L	−38	−75	−26	3.0
Inferior temporal gyrus	126	R	54	−9	−42	3.2
Pole of middle temporal gyrus	125	L	−42	3	−25	3.3

The table reports cluster size (in voxels), hemisphere (L, left; R, right), MNI coordinates (x, y, z) of the peak voxel, and corresponding *t*-values (*t*-stat).

## 4 Discussion

This proof-of-concept fMRI study provides initial evidence suggesting that the after-feel of a cosmetic formulation on the skin may influence the neural processing of affective touch. Our results indicate that after-feel is associated with modulation of brain activity extending beyond primary somatosensory regions to include areas implicated in reward and affective processing. Enhanced responses in the nucleus accumbens and amygdala suggest that formulation-related changes in skin texture and softness may contribute to the hedonic appraisal of tactile input, particularly in relation to the residual sensations experienced by the receiving hand during the after-feel phase. Notably, these neural effects were observed with cream A, providing direct evidence that formulation and ingredient composition can shape tactile-driven brain responses, beyond motor execution alone.

During the after-feel, residual tactile sensations experienced by the left (receiving) hand following self-touch produced higher activation in motor and somatosensory regions following cream application than in the no-cream condition, with cream A eliciting the greatest activation. Because tactile stimulation was self-generated, bilateral activation across sensorimotor cortices and the cerebellum is expected, reflecting the combination of motor commands from the active hand (right hand applying the cream) and tactile reafference from the stimulated hand (e.g., left hand). This bilateral sensorimotor pattern was observed consistently across all three conditions, confirming that movement execution and basic touch perception were comparable regardless of cream type. However, only cream A recruited regions beyond this shared sensorimotor baseline, most notably ventral prefrontal, orbitofrontal, and subgenual cingulate cortices. This suggests that while self-touch–related sensorimotor processing was present in all conditions, cream A specifically modulated the evaluative and affective processing of residual tactile sensations associated with the after-feel experience.

Consistent with these observations, and in line with findings by Querleux et al. (1999), our results support the view that alterations in skin surface properties induced by cosmetic application modulate somatosensory cortical responses in the skin receiving the tactile input. More recently, Bennett-Kennett et al. (2023) showed that topical products can induce cutaneous biomechanical changes that contribute to sensory perception, notably through the activation of low-adaptive afferent neurons associated with Merkel cell mechanoreceptors. These findings, based on correlations between perceived skin tightness and neural responses in a skin model, highlight the role of the topographical features of the skin surface in the propagation of strain to depth of mechanoreceptors, thereby enabling the perception of skin comfort or discomfort (Bennett-Kennett et al., 2023). The absorption of the cream’s ingredients, which depends on molecular weight, particle size, and lipophilicity (log P), influences skin surface characteristics and tactile perception (Förster et al., 2009). Accordingly, in the present proof-of-concept study, differences in emulsifier composition are likely to have modulated skin surface texture. Specifically, the micrometric (1–3 μm) polyhedral starch particles in cream A do not penetrate the skin but remain trapped within the skin microrelief after product penetration and drying. In contrast, the reference cream contains a lipid-based emulsifier with a lower molecular weight ( < 1,000 g/moL), which is expected to blend with and penetrate the upper layers of the stratum corneum. A previous study by Moussour et al. (2017) demonstrated that emulsions containing 5.5% of various powders, including starches, significantly enhanced the sensation of smoothness during the after-feel phase, an effect attributed to particles filling and smoothing surface irregularities of the skin. A roll-on effect, hypothesized to result from the physical movement of particles during stroking, may further reinforce these sensory attributes (Moussour et al., 2017). The perception of innocuous touch relies on low-threshold mechanoreceptors (LTMRs) mediated by Aβ fibers (Abraira and Ginty, 2013). Such discriminative processing appears to be particularly engaged by cream A, which elicited stronger bilateral somatosensory responses in primary and secondary somatosensory cortices. Enhanced smoothness associated with starch particles may contribute to perceived softness by preferentially engaging slowly adapting type 1 (SA1) mechanoreceptors (Condon et al., 2014). In addition, rapidly adapting mechanoreceptors sensitive to dynamic stimulation are likely engaged during tactile exploration. As Pacinian corpuscles are tuned to fine textures with spatial periods below 10 μm (Miyaoka, 2008), they may be especially responsive to the relief or rolling action of starch particles, thereby enhancing the tactile salience of after-feel sensations. Beyond discriminative touch processing, the sensation of softness typical of native starch powders (Olivato, 2024) represents an important attribute for cosmetic product appeal and may influence affective touch perception. Returning to the distinct pathways of pleasant touch and affective evaluation, activation of prefrontal regions and the amygdala (Gordon et al., 2013) support the view that the CT system is likely engaged when considering the passive reception of touch on the dorsum of the hand. Notably, the spatial and temporal features of self-touch in this paradigm, including a stroking velocity of approximately 9 cm/s, fall within the optimal range for CT fiber activation (Löken et al., 2009; Morrison, 2012). The activation observed in the posterior insula in both simple and complex contrasts further supports this interpretation (Björnsdotter et al., 2009).

In this context, the softness represents a salient tactile property of cream A, shaping residual skin sensations during the after-feel phase. On this sensory basis, cream A elicited a broader network of activations, extending beyond primary sensory and motor regions to include reward-related and higher-order cortical areas. Among these, the putamen also emerged in a simple contrast, suggesting that the altered tactile experience associated with the after-feel rather than with active movement have engaged reward-related processes. This interpretation is consistent with previous findings showing a time-dependent increase in putamen activation during sustained touch (Sailer et al., 2016). Similarly, Mayorova et al. (2023) reported significant putamen responses following stimulation of glabrous skin on the sole of the foot, regardless of whether the stimulus was delivered by hand or with a feather. Furthermore, McCabe et al. (2008) found a correlation between activation in the ventral striatum, including the putamen, and subjective pleasantness ratings when participants were touched on the forearm with a “rich” versus a “thin” body cream. Although affective touch paradigms consistently recruit the ventral striatum—spanning the ventral caudate and putamen in addition to the nucleus accumbens (NAcc)—recent evidence points to a functional dissociation within this territory. In particular, the NAcc has emerged as the ventral striatal subregion most tightly implicated in reward appraisal and socio-affective relevance (Mielacher et al., 2024; Stevens et al., 2024). In line with this, we observed right-lateralized NAcc engagement during the after-feel phase following cosmetic cream application, consistent with tactile stimulation of the left (receiving) hand, suggesting that even subtle residual tactile sensations can recruit reward-related circuits, even in non-social, non-explicitly emotional contexts. Complementing this, we also saw activation in the amygdala; a region traditionally associated with threat detection but increasingly recognized for its role in processing positive affective stimuli (Sergerie et al., 2008, Bonnet et al., 2015). Previous work (Gordon et al., 2013; Croy et al., 2016) has shown that the amygdala responds not only to fear but also to pleasant inputs such as sweet tastes and caresses, and that olfactory cues can modulate touch and amygdala–somatosensory connectivity. Together, these findings suggest that both the nucleus accumbens (NAcc) and the amygdala integrate sensory input with emotionally salient cues during affective touch, particularly in the context of passive tactile reception during after-feel, even in the absence of explicit social interaction.

Building on this, the pleasant after-feel associated with cream A engaged networks beyond core reward- and emotion-related regions, suggesting modulation of sensory and evaluative integration rather than increased somatosensory responses alone. Consistent with this interpretation, the contrast between cream A and the reference formulation revealed significant activation in the posterior inferior temporal gyrus. This region of the lateral temporal cortex has been associated with the encoding of fine surface features (Grill-Spector and Weiner, 2014) and is located near areas involved in the affective dimension of touch (Davidovic et al., 2016), positioning it as a plausible contributor to the integration of sensory detail with hedonic evaluation. Extensive cerebellar recruitment further aligns with evidence for its role in sensorimotor integration and the modulation of emotional processing beyond motor coordination (Schmahmann, 2019), suggesting that tactile evaluation involves interactions between perceptual, evaluative, and regulatory systems. Overall, these findings indicate that pleasant touch as experienced through after-feel rather than active movement engages a distributed, context-sensitive network that shapes the neural representation of tactile input, highlighting its function as a modulatory signal rather than a simple sensory or reward cue.

While the present findings offer initial insights into how cosmetic after-feel can modulate affective touch processing at the neural level, several limitations should be considered when interpreting the results. First, tactile stimulation in this study was implemented as a standardized model—applied in a consistent manner to the hand—which may not fully capture the complexity of real-life touch interactions or self-initiated tactile exploration in which active and passive components may be more strongly dissociated. Second, although participants were familiarized with the application procedure outside and inside the MRI environment, applying the cream required hand movements that may have induced subtle head motion, potentially affecting the BOLD signal. Third, only one reference formulation cream (e.g., cream B) was used for comparison, limiting our ability to determine whether the observed effects are specific to cream A or reflect broader differences in formulation type. Fourth, although the creams differed in texture, no objective physical assessments or subjective ratings were collected to characterize these properties or include them as covariates in the GLM. This absence of behavioral and psychometric measures limits our ability to link the observed neural responses to participants’ perceptual experience—particularly interpretations related to pleasantness or “liking.” In addition, a separate consumer study was conducted in an independent sample of 100 French women using a 1-week home-use protocol and a self-administered online questionnaire (reported in the Supplementary Tables 4, 5 — Consumer Study). While this consumer study provides only subjective evaluations of Cream A without comparison to a benchmark product and was conducted outside the MRI cohort, it nevertheless, offers complementary information regarding the overall perceived pleasantness of the product. Finally, the sample size was modest, and replication in a larger cohort would strengthen the generalizability of these findings and clarify whether the observed effects extend to other cosmetic textures or application contexts.

## 5 Conclusion

Finally, the after-feel associated with one of the compounds (quinoa-derived starch powder) enhanced bilateral somatosensory responses and additionally recruited regions linked to affective valuation and reward processing, which were lateralized toward the hemisphere contralateral to the hand receiving the stimulation, indicating that the residual tactile experience was perceived as more pleasant. This affective modulation was accompanied by the involvement of prefrontal and associative cortices, suggesting that pleasant after-feel engages not only emotional mechanisms but also higher-order appraisal processes that shape the interpretation of the sensory input. Together, these findings point to a hierarchical organization in which subtle variations in the physical properties of the stimuli influence successive stages of tactile processing, from sensory encoding to hedonic relevance and ultimately to cognitive evaluation.

## Data Availability

The raw data supporting the conclusions of this article will be made available by the authors, without undue reservation.

## References

[B1] Abraira V. E. Ginty D. D. (2013). The sensory neurons of touch. *Neuron* 79 618–639. 10.1016/j.neuron.2013.07.051 23972592 PMC3811145

[B2] Akanny E. Kohlmann C. (2024). Predicting tactile sensory attributes of personal care emulsions based on instrumental characterizations: A review. *Intern. J. Cosmet. Sci.* 46 1035–1063. 10.1111/ics.13004 39049783

[B3] Alcalá-López D. Mei N. Margolles P. Soto D. (2024). Brain-wide representation of social knowledge. *Soc. Cogn. Affect. Neurosci.* 19:nsae032. 10.1093/scan/nsae032 38804694 PMC11173195

[B4] Baki G. Szoboszlai M. Liberatore M. W. Chandler M. (2018). Application of Check-All-That-Apply (CATA) questions for sensory characterization of cosmetic emulsions by untrained consumers. *J. Cosmet. Sci.* 69 83–100. 29799807

[B5] Bennett-Kennett R. Pace J. Lynch B. Domanov Y. Luengo G. S. Potter A. (2023). Sensory neuron activation from topical treatments modulates the sensorial perception of human skin. *PNAS Nexus* 2:gad292. 10.1093/pnasnexus/pgad292 37771342 PMC10531117

[B6] Björnsdotter M. Löken L. Olausson H. Vallbo A. Wessberg J. (2009). Somatotopic organization of gentle touch processing in the posterior insular cortex. *J. Neurosci.* 29 9314–9320. 10.1523/JNEUROSCI.0400-09.2009 19625521 PMC6665561

[B7] Bonnet L. Comte A. Tatu L. Millot J.-L. Moulin T. Medeiros (2015). The role of the amygdala in the perception of positive emotions: An “intensity detector.”. *Front. Behav. Neurosci.* 9:178. 10.3389/fnbeh.2015.00178 26217205 PMC4493392

[B8] Condon M. Birznieks I. Hudson K. Chelvanayagam D. K. Mahns D. Olausson H. (2014). Differential sensitivity to surface compliance by tactile afferents in the human finger pad. *J. Neurophysiol.* 111 1308–1317. 10.1152/jn.00589.2013 24371291

[B9] Courrèges S. Aboulaasri R. Bhatara A. Bardel M.-H. (2021). Crossmodal interactions between olfaction and touch affecting well-being and perception of cosmetic creams. *Front. Psychol.* 12:703531. 10.3389/fpsyg.2021.703531 34484055 PMC8414979

[B10] Croy I. Drechsler E. Hamilton P. Hummel T. Olausson H. (2016). Olfactory modulation of affective touch processing — A neurophysiological investigation. *NeuroImage* 135 135–141. 10.1016/j.neuroimage.2016.04.046 27138206

[B11] Davidovic M. Jönsson E. H. Olausson H. Björnsdotter M. (2016). Posterior superior temporal sulcus responses predict perceived pleasantness of skin stroking. *Front. Hum. Neurosci.* 10:432. 10.3389/fnhum.2016.00432 27679564 PMC5020046

[B12] de Noronha R. L. F. Benson H. A. E. Leite-Silva V. R. (2019). “Sensory analysis applied to cosmetic products,” in *Cosmetic Formulation.* Boca Raton, FL: CRC Press. 10.1201/9780429190674-24

[B13] Duncan K. K. Nagashima M. Saheki Y. Tagai K. Shigemasu H. Kanayama. (2020). “Neuroscientific evidence that texture is multimodal and why that’s important for cosmetics,” in *Poster Presented at the Conference of the International Federation of Societies of Cosmetic Chemists*, (Hiratsuka: Yokohama).

[B14] Duncan K. Tokuda T. Sato C. Tagai K. Dan I. (2019). Willingness-to-Pay-Associated right prefrontal activation during a single, real use of cosmetics as revealed by functional near-infrared spectroscopy. *Front. Hum. Neurosci.* 13:16. 10.3389/fnhum.2019.00016 30778292 PMC6369365

[B15] Ebisch S. J. H. Ferri F. Salone A. Perrucci M. G. D’Amico L. Ferro F. M. (2011). Differential involvement of somatosensory and interoceptive cortices during the observation of affective touch. *J. Cogn. Neurosci.* 23 1808–1822. 10.1162/jocn.2010.21551 20666597

[B16] Esteban O. Markiewicz C. J. Blair R. W. Moodie C. A. Isik A. I. Erramuzpe A. (2019). fMRIPrep: A robust preprocessing pipeline for functional MRI. *Nat. Methods* 16 111–116. 10.1038/s41592-018-0235-4 30532080 PMC6319393

[B17] Förster M. Bolzinger M.-A. Fessi H. Briançon S. (2009). Topical delivery of cosmetics and drugs. Molecular aspects of percutaneous absorption and delivery. *Eur. J. Dermatol.* 19 309–323. 10.1684/ejd.2009.0676 19443302

[B18] Gabriel D. Merat E. Jeudy A. Cambos S. Chabin T. Giustiniani J. (2021). Emotional effects induced by the application of a cosmetic product: A real-time electrophysiological evaluation. *Appl. Sci.* 11:4766. 10.3390/app11114766

[B19] Gordon I. Voos A. C. Randi H. Bennet, Bolling D. Z. Pelphrey K. A. (2013). Brain mechanisms for processing affective touch. *Hum. Brain Mapp.* 34 914–922. 10.1002/hbm.21480 22125232 PMC6869848

[B20] Grill-Spector K. Weiner K. S. (2014). The functional architecture of the ventral temporal cortex and its role in categorization. *Nat. Rev. Neurosci.* 15 536–548. 10.1038/nrn3747 24962370 PMC4143420

[B21] Hirao N. Noriuchi M. Isobe H. Kikuchi Y. (2020). Luxury cues facilitate the connection between social dominance and reward mediated by the lateral prefrontal cortex. *J. Cosmet Sci.* 71 37–45. 32271707

[B22] Hirao N. Noriuchi M. Isobe H. Kikuchi Y. (2021). Luxury cues of cream heighten the reward value of its tactile experience. *J. Cosmet. Sci.* 72 81–89. 35349427

[B23] Johnson K. O. (2001). The roles and functions of cutaneous mechanoreceptors. *Curr. Opin. Neurobiol.* 11 455–461. 10.1016/s0959-4388(00)00234-8 11502392

[B24] Kogler L. Müller V. I. Werminghausen E. Eickhoff S. B. Derntl B. (2020). Do I feel or do I know? Neuroimaging meta-analyses on the multiple facets of empathy. *Cortex* 129 341–355. 10.1016/j.cortex.2020.04.031 32562973 PMC7390692

[B25] Krishna A. Luangrath A. W. Peck J. (2024). A review of touch research in consumer psychology. *J. Consum. Psychol.* 34 359–381. 10.1002/jcpy.1413

[B26] Lindgren L. Westling G. Brulin C. Lehtipalo S. Andersson M. Nyberg L. (2012). Pleasant human touch is represented in pregenual anterior cingulate cortex. *Neuroimage* 59 3427–3432. 10.1016/j.neuroimage.2011.11.013 22100768

[B27] Löken L. S. Wessberg J. Morrison I. McGlone F. Olausson H. (2009). Coding of pleasant touch by unmyelinated afferents in humans. *Nat. Neurosci.* 12 547–548. 10.1038/nn.2312 19363489

[B28] Lombardi S. A. (2017). Emotional effects induced by lip balms containing different emollients: Neuroscientific approach to studying the tactual experience. *Household Personal Care Today* 12 52–57.

[B29] Marshall A. G. McGlone F. P. (2020). Affective touch: The enigmatic spinal pathway of the C-Tactile afferent. *Neurosci. Insights* 15:2633105520925072. 10.1177/2633105520925072 32529186 PMC7265072

[B30] Mayorova L. Portnova G. Skorokhodov I. (2023). Cortical response variation with social and non-social affective touch processing in the glabrous and hairy skin of the leg: A pilot fMRI study. *Sensors* 23:7881. 10.3390/s23187881 37765936 PMC10538157

[B31] McCabe C. Rolls E. T. Bilderbeck A. McGlone F. (2008). Cognitive influences on the affective representation of touch and the sight of touch in the human brain. *Soc. Cogn. Affect. Neurosci.* 3 97–108. 10.1093/scan/nsn005 19015100 PMC2555465

[B32] McGlone F. Wessberg J. Olausson H. (2014). Discriminative and affective touch: Sensing and feeling. *Neuron* 82 737–755. 10.1016/j.neuron.2014.05.001 24853935

[B33] Mielacher C. Scheele D. Kiebs M. Schmitt L. Dellert T. Philipsen A. (2024). Altered reward network responses to social touch in major depression. *Psychol. Med.* 54 308–316. 10.1017/s0033291723001617 37272345

[B34] Miyaoka T. (2008). Submicron-texture-discrimination mechanisms in human tactile perception. *Proc. Fechner Day* 24, 151–156.

[B35] Morrison I. (2012). CT afferents. *Curr. Biol.* 22 R77–R78. 10.1016/j.cub.2011.11.032 22321302

[B36] Moussour M. Lavarde M. Pensé-Lhéritier A.-M. Bouton F. (2017). Sensory analysis of cosmetic powders: Personal care ingredients and emulsions. *Int. J. Cosmet. Sci.* 39 83–89. 10.1111/ics.12352 27383046

[B37] Olivato J. B. (2024). “Chapter 11 - Starch: A natural, safe, and multifunctional ingredient for cosmetic formulations,” in *Starch Industries: Processes and Innovative Products in Food and Non-Food Uses*, eds Cereda M. P. Vilpoux O. F. (Cambridge, MA: Academic Press).

[B38] Perini I. Morrison I. Olausson H. (2015). Seeking pleasant touch: Neural correlates of behavioral preferences for skin stroking. *Front. Behav. Neurosci.* 9:8. 10.3389/fnbeh.2015.00008 25698948 PMC4318429

[B39] Querleux B. Gazano G. Mohen-Domenech O. Jacquin J. Burnod Y. Gaudion P. (1999). Brain activation in response to a tactile stimulation: Functional magnetic resonance imaging (FMRI) versus cognitive analysis. *Int. J. Cosmet. Sci.* 21 107–118. 10.1046/j.1467-2494.1999.198270.x 18505535

[B40] Rolls E. T. O’Doherty J. Kringelbach M. L. Francis S. Bowtell R. McGlone F. (2003). Representations of pleasant and painful touch in the human orbitofrontal and cingulate cortices. *Cereb. Cortex* 13 308–317. 10.1093/cercor/13.3.308 12571120

[B41] Sailer U. Triscoli C. Häggblad G. Hamilton P. Olausson H. Croy I. (2016). Temporal dynamics of brain activation during 40 minutes of pleasant touch. *NeuroImage* 139 360–367. 10.1016/j.neuroimage.2016.06.031 27338514

[B42] Savary G. Gilbert L. Grisel M. Picard C. (2019). Instrumental and sensory methodologies to characterize the residual film of topical products applied to skin. *Skin Res. Technol.* 25 415–423. 10.1111/srt.12667 30767275

[B43] Schmahmann J. D. (2019). The cerebellum and cognition. *Neurosci. Lett.* 688 62–75. 10.1016/j.neulet.2018.07.005 29997061

[B44] Sergerie K. Chochol C. Armony J. L. (2008). The role of the amygdala in emotional processing: A quantitative meta-analysis of functional neuroimaging studies. *Neurosci. Biobehav. Rev.* 32 811–830. 10.1016/j.neubiorev.2007.12.002 18316124

[B45] Shirata M. M. F. Campos P. M. B. G. M. (2016). Importance of texture and sensorial profile in cosmetic formulations development. *Surg. Cosmet. Dermatol.* 8 223–230. 10.5935/scd1984-8773.201683861

[B46] Spence C. Zhang T. (2024). Multisensory contributions to skin-cosmetic product interactions. *Intern. J. Cosmet. Sci.* 46 833–849. 10.1111/ics.12975 38761125

[B47] Stevens L. Bregulla M. Scheele D. (2024). Out of touch? How trauma shapes the experience of social touch – Neural and endocrine pathways. *Neurosci. Biobehav. Rev.* 159:105595. 10.1016/j.neubiorev.2024.105595 38373642

[B48] Wang F. Ma X. Cheng D. Gao L. Yao C. Lin W. (2024). Electroencephalography as an objective method for assessing subjective emotions during the application of cream. *Skin Res. Technol.* 30:e13692. 10.1111/srt.13692 38650354 PMC11035903

