# Supplementary material for: Cosmetic after-feel modulates brain activity in sensory and reward networks: an fMRI study
Source: Front Neurosci. 2026 Mar 10;20:1759372. doi: 10.3389/fnins.2026.1759372 (PMC13008859; doi:10.3389/fnins.2026.1759372)
Supplement: Supplementary file 1 [file Data_Sheet_1.docx]

**SUPPLEMENTS**

|  | | **Cream A**  **Test Emulsion** | **Cream B**  **Reference emulsion** |
| --- | --- | --- | --- |
| **Ingredient** | **INCI name** | **(%)** | **(%)** |
| Deionized Water | Water | 71,95 | 72,95 |
| Preservative system | Pentylene Glycol (and) glyceryl caprylate/caprate | 2,5 | 2,5 |
| Xanthan gum | Xanthan gum | 0.50 | 0.50 |
| Pickering emulsifier | Sodium starch Octenylsuccinate | 5.00 | 0.00 |
| Emulsifier Benchmark | Glyceryl stearate (and) PEG-100 stearate | 0.00 | 4.00 |
| Sweet almond oil | Prunus amygalus dulcis (sweet almond) oil | 14.00 | 14.00 |
| Shea butter | Shea butter ethyl esters | 3.00 | 3.00 |
| Coco-caprylate | Coco-caprylate | 3.00 | 3.00 |
| Anti-oxidant | Tocopherol(and)helianthus annuus (sunflower) oil | 0.05 | 0.05 |

**Supplementary Table I. Cream A and cream B ingredient composition.**

**
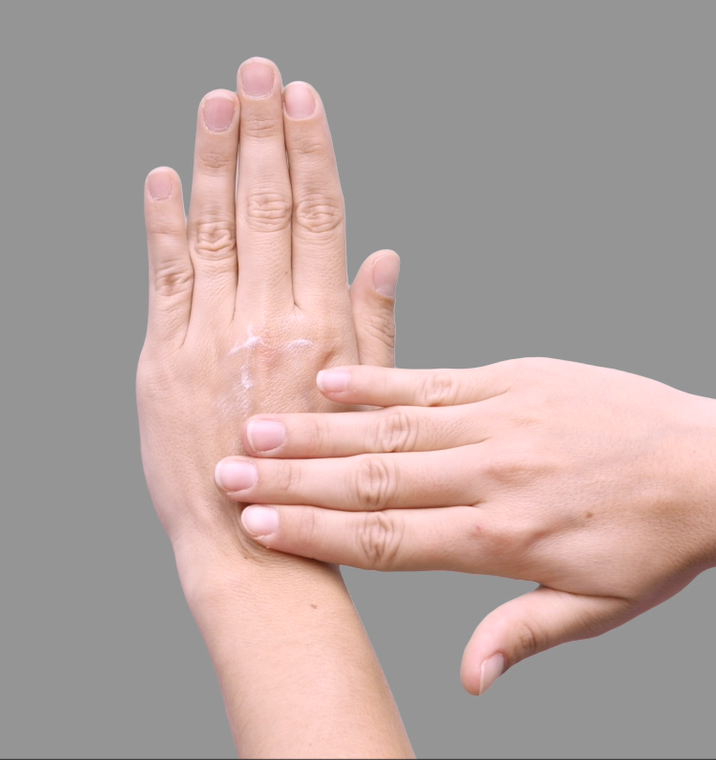
**

**Supplementary Image 1. Image captured from the training video .** Prior to the session, this video showing the application and the after-feel movement was watched by the panelist to ensure the number of rotations, the speed ( 2s/ rotation), and the right trajectory.

**Simple contrast**

| **Structures** | **Size** | **Side** | **MNI Coordinates** | | |  |
| --- | --- | --- | --- | --- | --- | --- |
|  |  |  | ***x*** | ***y*** | ***z*** | ***t-stat*** |
| Postcentral gyrus | 6661 | L | -44 | -21 | 64 | 5.6 |
| Cerebellum | 707 | R | 14 | -50 | -18 | 4.2 |

**Supplementary Table 2. Brain regions showing significant activation for the simple contrast *no cream > baseline.*** The table lists cluster size (in voxels), hemisphere (L: left; R: right), MNI coordinates (x, y, z) of the peak voxel, and the corresponding t-value (t-stat).


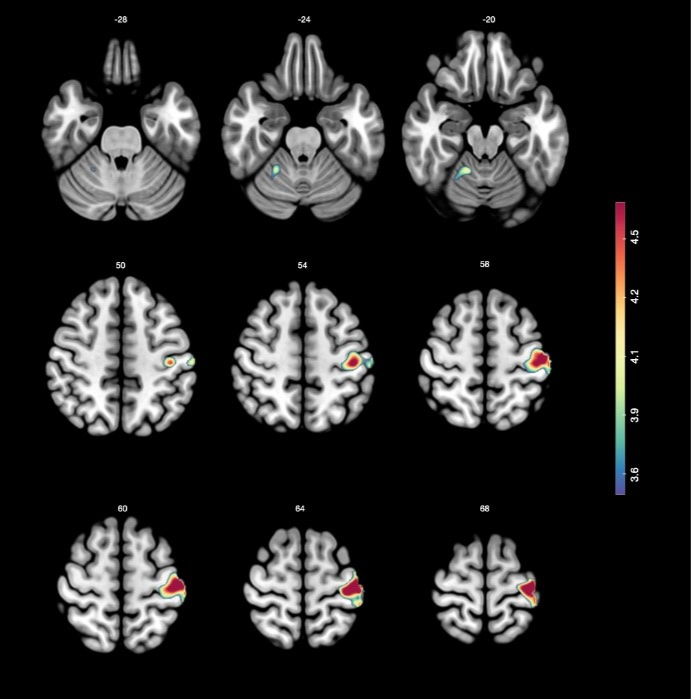

**Supplementary Figure 1. Brain activation map for the contrast *no cream > baseline*.** Statistical parametric map showing significant activation associated with the no cream condition compared to baseline. Axial slices are displayed at multiple MNI z-coordinates (z = –28 to 68). The color scale indicates t-values from the group-level analysis, thresholder at p < 0.05 (FDR-corrected).

| **Structures** | **Size** | **Side** | **MNI Coordinates** | | |  |
| --- | --- | --- | --- | --- | --- | --- |
|  |  |  | ***x*** | ***y*** | ***z*** | ***t-stat*** |
| Postcentral gyrus | 12413 | L | -48 | -17 | 60 | 5.8 |
| Cerebellum | 1884 | R | 26 | 56 | -22 | 4.1 |
|  | 859 | R | 22 | -69 | -48 | 4.3 |
| Precentral gyrus | 459 | L | -6 | -13 | 52 | 4.0 |

**Supplementary Table 3. Brain regions showing significant activation for the simple contrast *cream B > baseline.*** The table reports cluster size (in voxels), hemisphere (L: left; R: right), MNI coordinates (x, y, z) of the peak voxel, and corresponding t-values (t-stat).


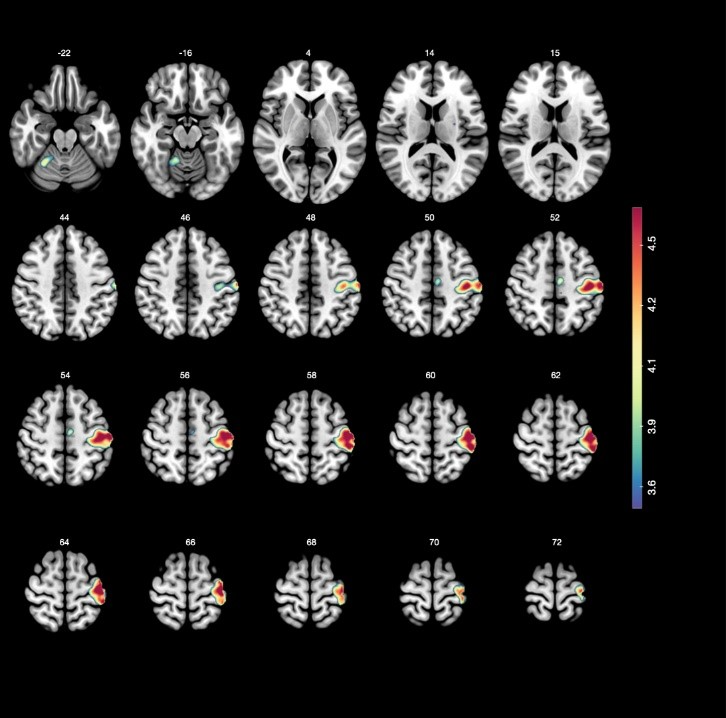

**Supplementary Figure 2. Brain activation map for the contrast *cream B > baseline****.* Statistical parametric map showing significant activation associated with the cream B condition compared to baseline. Axial slices are displayed at multiple MNI z-coordinates (z = –22 to 72). The color scale indicates t-values from the group-level analysis, thresholder at p < 0.05 (FDR-corrected).

**Supplementary materials-Consumer Study**

| Consumer study | Total agree % (strongly agree + rather agree) |
| --- | --- |
| Visual aspect | **Cream A** |
| The very white aspect of this lotion is very pleasing | 89% |
| The very white and shiny appearance of this cream is particularly attractive | 86% |
|  |  |
| Application | **Cream A** |
| Upon application, the cream has a very pleasant soft and powdery finish | 86% |
| I love how soft my skin feels after applying the cream | 87% |
| I like that the cream fades, its evanescent side | 88% |
| The soft, powdery and light touch of this product is quite unique | 79% |
|  |  |
| Consumer appreciation | **Cream A** |
| The cream makes me feel good | 77% |
| The cream makes me feel good about myself | 71% |
| My skin loved this cream | 83% |
| This cream respects the balance of my skin | 81% |
|  |  |
| Comparison with the usual product | **Cream A** |
| The very white and shiny appearance of the cream is more attractive than the visual appearance of my usual product | 71% |
| The application of this product is more pleasant than that with my usual product | 65% |
| Using this product brings me more well-being than my usual product | 56% |

**Supplementary Table 4. Results of the consumer study conducted with cream A (oil-in-water (O/W) emulsion containing 5% modified quinoa starch).** One hundred French women (50% aged 18–40 years and 50% aged 41–65 years), regular users of face cream (≥5 times per week), participated in a one-week home-use test replacing their usual products. Consumer perceptions were collected via a self-administered online questionnaire. Data are expressed as the percentage of respondents who totally agree and rather agree with each statement, covering visual aspect, application properties, overall consumer appreciation, and comparison with the usual product.

| Global liking | Cream A | |
| --- | --- | --- |
|  | Dislike Extremely | 0% |
|  | Dislike Very Much | 0% |
|  | Dislike Moderately | 0% |
|  | Dislike Slightly | 4% |
|  | Neither Like nor Dislike | 3% |
|  | Like Slightly | 7% |
|  | Like Moderately | 30% |
|  | Like Very Much | 34% |
|  | Like Extremely | 22% |
| Texture application | **Cream A** |  |
|  | Very unpleasant | 0% |
|  | Rather unpleasant | 4% |
|  | Neither pleasant, nor u | 5% |
|  | Rather pleasant | 41% |
|  | Very pleasant | 50% |

**Supplementary Table 5. Consumer global liking and texture application evaluation of the face cream A.** Distribution of consumer responses for global liking and texture application pleasantness of the face cream containing 5% modified quinoa starch after one week of home use. Global liking was assessed using a 9-point hedonic scale ranging from *“Dislike extremely”* to *“Like extremely”*. Texture application pleasantness was evaluated using a 5-point scale ranging from *“Very unpleasant”* to *“Very pleasant”*. Results are expressed as percentages of consumers.


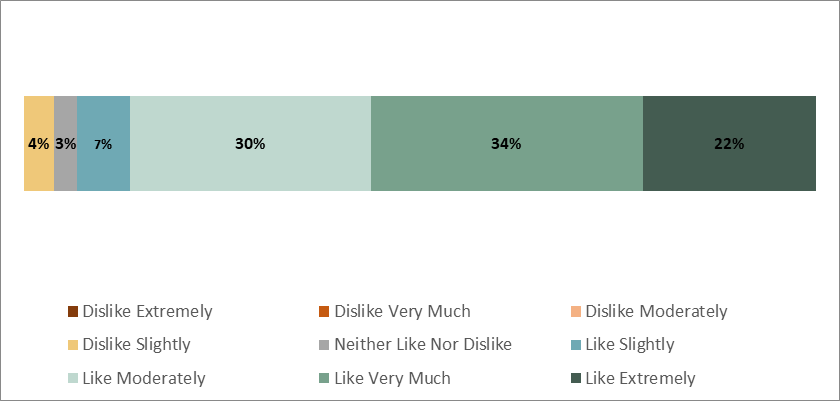


**Supplementary Figure 3. Consumer evaluation of texture application pleasantness for the face cream A.** Responses were collected on a 5-point scale from “Very unpleasant” to “Very pleasant” and are presented as percentages of consumers.
